# *Clostridium difficile* infection in the Lao People’s Democratic Republic: first isolation and review of the literature

**DOI:** 10.1186/s12879-017-2737-6

**Published:** 2017-09-21

**Authors:** Elaine Cheong, Tamalee Roberts, Sayaphet Rattanavong, Thomas V. Riley, Paul N. Newton, David A. B. Dance

**Affiliations:** 1 Lao-Oxford-Mahosot Hospital-Wellcome Trust Research Unit, Microbiology Laboratory, Mahosot Hospital, Vientiane, Lao People’s Democratic Republic; 2 Department of Microbiology, Concord Repatriation General Hospital, Sydney, Concord Australia; 3 PathWest Laboratory Medicine (WA), Edith Cowan University and Murdoch University, Queen Elizabeth II Medical Centre, Nedlands, Australia; 4 Centre for Tropical Medicine & Global Health, University of Oxford, Oxford, UK; 5 Faculty of Infectious and Tropical Diseases, London School of Hygiene and Tropical Medicine, London, UK

**Keywords:** *Clostridium Difficile*, Lao PDR, Laos, Antibiotic associated diarrhoea, Ribotypes

## Abstract

**Background:**

Current knowledge of the epidemiology of *Clostridium difficile* infection in Asia, and in particular the Greater Mekong Subregion, is very limited. Only a few studies from Thailand and Vietnam have been reported from the region with variable testing methods and results, and no studies from Lao People’s Democratic Republic (PDR). Therefore we investigated the presence of *C. difficile* in a single centre in the Lao PDR and determined the ribotypes present.

**Method:**

Seventy unformed stool samples from hospital inpatients at Mahosot Hospital, Vientiane, were tested for the presence of *C. difficile* using selective differential agar and confirmed by latex agglutination. *C. difficile* isolates were further characterised by ribotyping and toxin gene detection.

**Results:**

*C. difficile* was isolated from five of the 70 patients, and five different ribotypes were identified (014, 017, 020, QX 107 and QX 574).

**Conclusion:**

This is the first isolation of *C. difficile* from human stool samples in the Lao PDR. These results will add to the limited amount of data on *C. difficile* in the region. In addition, we hope this information will alert clinicians to the presence of *C. difficile* in the country and will help inform future investigations into the epidemiology and diagnosis of *C. difficile* in Lao PDR.

## Background

*Clostridium difficile* is an anaerobic, Gram-positive, spore forming bacterium that causes antibiotic-associated diarrhoea which can vary from mild and self-limiting to serious manifestations including pseudomembranous colitis [1]. *C. difficile* infection (CDI) is predominantly healthcare-associated although increasing numbers of community-acquired infections have been reported [2, 3]. Some strains of *C. difficile* have the ability to produce three toxins - toxin A, toxin B and a binary cytolethal distending toxin (CDT), and only strains that can produce at least one of these toxins cause disease [4].

*C. difficile* is widely recognized as an important diarrheal pathogen in North America and Europe. Epidemics have occurred in both these regions, resulting in the development of guidelines on active surveillance of infection, laboratory diagnosis, management and infection prevention and control, and a need for better antibiotic stewardship within health care facilities, the community and animal production facilities [5]. In contrast, limited information on CDI is available in Asia [6]. Reports from the region are highly variable with respect to the prevalence, laboratory diagnostic methods and circulating ribotypes (RT). Not all countries in the region have reported CDI, and comparisons within the region are difficult to make [6].

Despite a number of reports of CDI in the neighbouring countries of Thailand [7] and China [8], there have been no published reports on the isolation of *C. difficile* in Lao People’s Democratic Republic (Lao PDR, Laos). Cephalosporins, particularly ceftriaxone, are used extensively in hospitals in Laos, which may carry an associated risk of CDI. We therefore investigated the prevalence of *C. difficile* from stool samples from hospitalised patients from a single centre within Laos and characterised all *C. difficile* isolates.

## Methods

The investigation was conducted in the Microbiology Laboratory at Mahosot Hospital during September and October 2013. Mahosot Hospital is a 350-bed government hospital in the Lao capital, Vientiane, which houses a range of medical and surgical units. There were 21,549 people admitted to the hospital during 2013 and 3540 people admitted during the months of September and October.

Routine testing of faeces in the Mahosot Hospital Microbiology Laboratory consists of microscopy for white cells, and culture for *Salmonella* and *Shigella* species only. *Vibrio* species are sought using selective media (thiosulfate-citrate-bile salts-sucrose agar; TCBS agar) when indicated by clinical or epidemiological features. The laboratory does not routinely undertake any anaerobic culture or attempt to detect *Campylobacter* species or viral enteric pathogens, whilst microscopy for parasites is conducted in a separate laboratory. Relatively small numbers of stool samples are sent to the laboratory. However, because of concern that *C. difficile* may be unrecognised in Laos, methods for *C. difficile* culture and identification were established as part of routine stool examination for a 2 month period. Patients gave oral consent for the collection of stool samples for diagnosis of their illness.

A memo (in Lao language) was sent to the medical teams in Mahosot Hospital notifying them of the introduction of *C. difficile* diagnostic techniques. The following details were obtained for each specimen: date of specimen collection, patient age and gender, clinical diagnosis as listed on the test request form, the presence of stool white cells on microscopy and the results of *Salmonella*/*Shigella* culture. Other microbiologic details were noted if present; e.g. the presence of faecal parasites on microscopy. Unfortunately it was not possible to obtain a reliable history for prior antibiotic use for every patient.

Unformed faecal specimens were directly plated onto half plates of selective differential medium (ChromID C. difficile agar, bioMerieux, France) and streaked for single colonies. Plates were incubated anaerobically (BD GasPak™ EZ Anaerobic Container System, Becton Dickinson, USA) for 48 h before examination for colonies typical of *C. difficile* (black, spreading, medusa-head colonies with a characteristic odour). Suspicious colonies were tested by latex agglutination according to the manufacturer’s instructions (Oxoid *C. difficile* latex test kit, Oxoid, UK) to confirm their identity. A control strain of *C. difficile* (*C. difficile* ATCC 9689) was used to validate test results. Isolates were stored at −80 °C until shipment to Perth, Australia, for confirmation of identity, testing for the presence of toxin genes (*tcdA*, *tcdB*, and *cdt*) and ribotyping as described previously targeting the 16S–23S rRNA gene using primers 5′-CGTGGGGTGAAGTCGTAACAAGG-3′ (positions 1445 to 1466 of the 16S rRNA gene) and 5′-GCGCCCTTTGTAGCTTGACC-3′ (positions 20 to 1 of the 23S rRNA gene) [9]. Antimicrobial susceptibility testing was also performed for moxifloxacin, metronidazole, clindamycin and vancomycin using E-tests (Biomérieux, Marcy-l’Etoile, France) with minimum inhibitory concentration (MIC) results interpreted according to current US Clinical and Laboratory Standards Institute (CLSI) guidelines.

## Results

A total of 86 faecal samples were received during the 2 month period, 70 of which were available for *C. difficile* culture. The median age was 35 years with a range of 240 days to 89 years. There were 31 females and 39 males. Of the 70 patients included in the study, 26 were reported to have diarrhoea, 14 had suspected typhoid fever, five had fever of unknown etiology and two had suspected melioidosis. *Salmonella spp.* were isolated from six of the 70 samples and five were positive for *C. difficile* by culture and latex agglutination. Three of the five isolates of *C. difficile* were positive for the *tcdB* gene with two also *tcdA* positive by polymerase chain reaction (PCR). There were five different RTs identified-RTs 014, 017, 020, QX 107 and QX 574, with the latter two isolates being non-toxigenic (Table 1). All isolates were susceptible to moxifloxacin, metronidazole and vancomycin. Four of the five isolates were clindamycin intermediate and isolate the RT QX107 isolate was clindamycin resistant with an MIC of 8 μg/ml.

**Table 1:** *Clostridium difficile* positive patients at Mahosot Hospital

Case	Age (Years)	Toxin gene profile	Ribotype	Stool culture	Clinical details	Prior antibiotic use
1	35	A-B-CDT-	QX 574	Negative	Family contact of *S*. *typhi* patient	Unknown
2	46	A + B + CDT-	UK 020	*Salmonella* sp.	Culture positive melioidosis, diarrhoea	Yes: ofloxacin 5 days
3	1	A-B + CDT-	UK 017	*Salmonella* sp.	Chronic diarrhoea	Yes: amoxicillin (unknown duration) ceftriaxone 3 days
4	1	A-B-CDT-	QX 107	Negative	Diarrhoea, 4–5 days	Unknown
5	7	A + B + CDT-	UK 014	Negative	Diarrhoea, unknown duration	Unknown

## Discussion

This is the first report, as far as we have been able to establish, of the isolation of *C. difficile* from diarrheal stool specimens in Laos. In a study of the etiology of diarrhoea in Vientiane from 1996 to 1997, the presence of *C. difficile* was not investigated [10], and there have been no clinical reports of CDI in Laos, of which awareness amongst doctors appears low. However, increasing awareness of this pathogen is leading to increased testing and improved surveillance elsewhere in Asia [6]. It is not possible to be certain that *C. difficile* was a primary pathogen in all five patients given the presence of *Salmonella* sp. in two patients and the absence of toxin genes in two of the *C. difficile* isolates. Furthermore, two of the isolates were from 1-year-old children. *C. difficile* is known to cause asymptomatic colonization in infants under 2 years of age, so the *C. difficile* in these two cases may not have been responsible for causing symptoms [11].

Although many studies have been published on *C. difficile* epidemiology worldwide, few are from Asia, and in particular the Greater Mekong Subregion. Reports from Thailand (Bangkok) predominate, with only a single report from Vietnam (Table 2). To date there are no published data on *C. difficile* infection and epidemiology from Laos, Cambodia or Myanmar showing the need for further research in these countries. The incidence of *C. difficile* infection in the different groups of patients listed in Table 2 ranged from 6.5% to 44%. Different diagnostic techniques were used for these studies, making comparisons difficult. Toxin enzyme immunoassay (EIA) tests alone, as used in two of these studies [12, 13], have a lower sensitivity compared to EIA paired with PCR [14, 15]. Three of the earliest Thailand studies also only looked at toxin A or the *tcdA* gene [12, 13, 16]. Due to the high regional prevalence of RT 017, which does not produce toxin A, it is possible that the true incidence of CDI has been underestimated in these studies [6].

**Table 2:** *Clostridium difficile* studies from the Greater Mekong Subregion

Location, Country	Number positive/number tested	Clinical presentation	Test method	Gene	Ribotypes (number positive)	Year of study	Reference
Bangkok, Thailand	123/279 (44%) (106/203 patients with diarrhoea 17/76 healthy controls)	Patients with diarrhoea and healthy controls. 84% of patients infants aged 0–3 years	Tissue culture cytotoxin assay			1990	[32]
Bangkok, Thailand	21/320 (6.5%) (15/140 clindamycin treated patients, 14/140 β-lactam-treated patients, 2/140 controls)	Antibiotic treated patients and healthy controls. All >15 years	Toxin A EIA (TechLab, BioWhittaker)			1991–1994	[12]
Bangkok, Thailand	77/443 (17.4%) (28/235 asymptomatic infants 16/76, asymptomatic children, 20/48 antimicrobial treated adults, 13/84 non-antimicrobial-treated adults)	Asymptomatic infants <12 months old, asymptomatic children 1–11 years old, antimicrobial treated diarrheal adults, non-antimicrobial treated diarrheal positive adults	Culture on cycloserine-cefoxitin-fructose agar, *tcdA* gene confirmed by in- house PCR	20 *tcdA* positive (2 from the infants and children group, 10 from antimicrobial treated adults and 8 from non-antimicrobial treated adults)		1998–1999	[16]
Bangkok, Thailand	140/472 (29.6%) (20/34 HIV-positive diarrheal patients, 21/167 HIV-positive non-diarrheal patients, 99/271 HIV-negative diarrhoeal patients)	HIV-positive diarrheal patients, HIV-positive non diarrheal patients and HIV-negative diarrheal patients	Cultured on cycloserine-cefoxitin-fructose agar (CCFA, Oxoid) CD-D1 latex kit (Mitsubishi Chemical Industries, Tokyo)			Unknown (published 2000)	[33]
Bangkok, Thailand	16/102 (15.6%)	HIV patients with diarrhoea	Toxin A EIA (Oxoid)			1999–2000	[13]
Bangkok, Thailand	53	Patients with suspected *C. difficile* infection	Qualitative immunochromatographic assay (Xpect C. difficile toxin A/B test; Thermo scientific, Lenexa, KS, USA), 5-plex PCR and an in-house PCR for the presence of *tcdA*	*tcdA, tcdB*	UK 017 (23), UK 014/020 (13), QX370 (1)	2006–2008	[17]
Bangkok, Thailand	25/203 (12.3%)	Diarrheal inpatients (>14 years old)	Immunochromatography (Remel Xpect)	*tcdA, tcdB*		2008	[34]
Bangkok, Thailand	47/175 (26.8%)	Hospital patients (≥15 years)	Toxin A/B by EIA (VIDAS; bioMerieux), *tcdB* by PCR	*tcdB*		2010–2011	[14]
Bangkok, Thailand	105/422 (24.9%)	Hospital patients with diarrhoea >18 years	Cultured on C. difficile ChromID agar (bioMérieux, Marcy l’Etoile, France), in-house PCRs for the presence of *tcdA* and *tcdB*, and binary toxin genes (*cdtA* and *cdtB*)	39 toxigenic- 27 *tcdA,* 12 *tcdB*	014/020 (17), 010 (12), 017 (12), 039 (9), 009 (6)	2015	[19]
Thailand	107/574 (18.6%)	Hospital patients with diarrhoea	EIA (Meridian Premier Cytoclone), PCR	48 *tcdA* and *tcdB* positive by PCR		Unknown (published 2003)	[35]
Vietnam	45/479 (9.4%)	Hospital patients with diarrhoea	Luminex xTAG gastrointestinal pathogen panel assay (Luminex Molecular Diagnostics, Austin, TX, USA)	30 *tcdA* and 15 *tcdB*		2009–2014	[36]

All of the ribotypes detected in this current study have been reported elsewhere. RTs 014 and 020 have been isolated in several studies from Thailand and China and were both isolated in this study [8, 17]. Due to the high similarity of these two RTs, they are often reported in studies as the ‘RT 014/020 group’. One RT 017 isolate was identified in our study. This is a prevalent RT in many countries in Asia and the predominant RT found in Thailand, China and South Korea [8, 17, 18]. Two isolates of RT QX 107 were recently described for the first time from Thailand [19] with our isolate being the third detected in the region. This could suggest that this RT is of Asian origin. The RT QX 574 isolated from this study may also be unique to the region, as the only other country the strain has been isolated from is Indonesia (T. Riley, unpublished data). There were no hypervirulent RT 027 or RT 078 isolated from our small number of patients. While both RT 027 and RT 078 have been widely reported from Europe and North America [20–22], there have only been sporadic reports from Asia, including recent reports from mainland China [18, 23, 24]. The spread of these RTs into mainland China could lead to further dissemination within South East Asia. RT 078 is more commonly associated with community-acquired *C. difficile* infection; hence hospital-based studies, as most of the studies in this region have been, might miss cases caused by this RT [25].

In Europe and the USA, the majority of *C. difficile* cases are thought to result from person-to-person spread. Antimicrobial resistant bacteria have become an established problem globally, and in particular in Asia, as a result of widespread overuse and misuse of antibiotics [26, 27]. This is also important in the promotion of antibiotic-associated diarrhoea and CDI. The use of clindamycin, third generation cephalosporins, penicillins and fluoroquinolones greatly increases the risk of CDI [28], with the third generation cephalosporins causing the highest attributable risk due to their frequent use in hospitals [29]. One study showed that two thirds of patients with CDI had received a cephalosporin during the two month period before diagnosis [30]. The use of cephalosporins, particularly ceftriaxone, is increasing in Laos, with a 50% increase in ceftriaxone usage seen in Mahosot Hospital since 2011 (Mahosot Hospital, unpublished data). Furthermore, nearly 50% of patients admitted to Mahosot Hospital have evidence of having received antibiotics prior to admission [27]. This highlights the need for enhanced antimicrobial stewardship if further increases in CDI are to be avoided.

Given the prevalence of factors which promote CDI, and the evidence that CDI occurs at similar rates in Asia as in other continents [6], we believe that CDI is likely to be an under-recognised cause of diarrhoea in Laos and adjoining countries. There are several reasons for this under-diagnosis, including low clinician awareness of CDI [31], limited access to diagnostic services, a disincentive to investigate diarrheal illnesses when the cost of testing is borne by the individual, and short hospital stays. These Lao results are limited by the small sample size, the sparse clinical information available, and the use of culture alone. However, we hope that this study will alert clinicians and policy makers to the presence of this bacterium in Laos. As in many parts of Asia, this country is experiencing rapid economic and demographic changes, and changes in healthcare which will further increase the potential importance of CDI. Enhanced surveillance will be required to fully appreciate the extent and impact of CDI in Laos, and elsewhere in the region.

## Conclusions

With the isolation of *C.difficile* from human diarrhoeal stool samples in Lao PDR, we can include this bacterium as a potential diarrhoeal pathogen in this country. This is an important first step in its recognition by clinicians and epidemiologists both within the country itself and the Greater Mekong Subregion of Asia. Future local investment in the laboratory diagnosis of CDI, the typing of isolates, and defining the clinical epidemiology of infection in the region is required to better inform healthcare providers in the development of clinical management algorithms, infection control and prevention practices, and policies for the prudent use of antimicrobials.

## Abbreviations

*CDI*: Clostridium difficile infection
*CDT*: Cytolethal distending toxin
*CLSI*: Clinical and Laboratory Standards Institute
*EIA*: Enzyme immunoassay
*Lao PDR*: Lao People’s Democratic Republic
*MIC*: Minimum inhibitory concentration
*PCR*: Polymerase chain reaction
*RT*: Ribotypes
*TCBS agar*: Thiosulfate-citrate-bile salts-sucrose agar
